# Effect of fatigue on quality of life in patients with rheumatoid arthritis: the chain mediating role of resilience and self-efficacy

**DOI:** 10.1186/s42358-024-00410-x

**Published:** 2024-09-09

**Authors:** Jian Zhou, Xinxin Fan, Yuqin Gan, Zongting Luo, Hong Qi, Yuqiong Cao

**Affiliations:** 1 Department of Orthopaedics, School of Clinical Medicine & The First Affiliated Hospital of Chengdu Medical College, Chengdu, China; 2 School of Nursing, Chengdu Medical College, Chengdu, China; 3 Department of Nursing, Chengdu Third People’s Hospital, Chengdu, China; 4 Department of Rheumatology and Immunology, Sichuan Academy of Medical Sciences & Sichuan Provincial People’s Hospital, 32# W. Sec 2, 1st Ring Rd, Chengdu, 610072 Sichuan Province China

**Keywords:** Rheumatoid arthritis, Fatigue, Quality of life, Resilience, Self-efficacy

## Abstract

**Objectives:**

Exploring the effect of resilience and self-efficacy in mediating the chain between fatigue and quality of life(QOL) in patients with rheumatoid arthritis (RA).

**Methods:**

From June 2022 to November 2022, 423 RA patients were chosen by a convenience sample method from two tertiary care facilities in Chengdu, Sichuan Province. General Information Questionnaire, Bristol Multidimensional Scale of Fatigue in Patients with Rheumatoid Arthritis, SF−12 Health Survey Short Form, Chinese version of the ten-item psychological Resilience Scale, and Chinese-language Arthritis Self-Efficacy Scale, an 8-element version, were among the questionnaires used.

**Results:**

In the physical component summary( PCS), self-efficacy, psychological resilience, and self-efficacy were all significantly mediated by fatigue (total effect mediated 8.88%). In the mental component summary (MCS), fatigue (total effect mediated 10.79%), self-efficacy (total effect mediated 8.99%), psychological resilience, and self-efficacy (total effect mediated 2.01%) were all significantly mediated by fatigue.

**Conclusion:**

Fatigue in RA patients can affect the quality of life both directly and indirectly through the mediating effects of psychological resilience, self-efficacy, and the chain mediating effect of psychological resilience-self-efficacy.

## Introduction

Rheumatoid arthritis (RA) is an autoimmune disorder that severely affects the musculoskeletal system and other underlying organs, resulting in joint deformity and a loss of function. The prevalence of RA is approximately 0.5–1% worldwide, and the number of people with RA in China exceeds 5 million [1, 2]. As the disease progresses, RA patients experience functional impairment, fatigue, and emotional disturbance, inflicting a high burden on them and reducing their quality of life (QOL) [3, 4]. Fatigue has a significant impact on quality of life and is present in over 70% of cases [5]. Patients with RA who experiences high levels of fatigue have a poor QOL and a high psychological burden [6]. Negative emotions are often the source of psychological burden, presenting a lack of robust resilience in the face of stress, resulting in poor resilience and an inability to cope well with stress caused by factors such as illness [7].

Resilience is the psychological potential of a person following a traumatic event and plays a crucial role in the development of self-efficacy and health behaviors [8, 9]. Researchers found that resilience positively correlated with QOL in RA patients [8], primarily because higher levels of resilience may help patients adapt to their condition and reinvent themselves, which is an important psychological resource for improving the QOL for RA patients. While self-efficacy influences the resilience of people with RA, the greater the self-efficacy, the higher the patients’ psychological resilience [10]. Self-efficacy refers to one’s belief or confidence to achieve behavioral goals in a specific domain [11]. According to self-efficacy theory, self-efficacy for health-affecting behaviors may predict future health status because individuals feel that the consequence of the behavior will improve their health status [11]. Studies have shown that the QOL of people with RA positively correlates with their self-efficacy [8, 12]. Based on the above discussion, it is clear that the negative emotions generated by fatigue symptoms in people with RA may reduce their resilience and self-efficacy [13], which impacts their QOL. Despite studies exploring fatigue and QOL in patients with RA, most have only conducted a single study of their correlates, with fewer studies analyzing pathways considering the potential role of fatigue, resilience, and self-efficacy on QOL in people with RA. Thus, the present study hypothesized that the resilience and self-efficacy of RA patients might serve as a chain mediator between fatigue and QOL. Using RA patients as the study population allowed us to examine the above hypothesis, investigate the pathways of the effects of fatigue, resilience, self-efficacy, and QOL, and provide the theoretical foundation and suggestions for improving the QOL of people with RA.

## Material and methods

### Clinical samples

This study was conducted on 423 patients with rheumatoid arthritis at two tertiary care hospitals in Chengdu. A convenience sampling approach was used to select Sichuan Province from June 2022 to November 2022. Inclusion criteria are as follows: (1) age ≥ 18 years; (2) satisfy the 2010 edition of the American College of Rheumatology and European League Against Rheumatism classification criteria for RA [14]; (3) consent to participate in this survey and sign an informed consent form; (4) complete the survey on their own. Exclusion criteria are as follows: (1) other diseases that severely affect the QOL, such as heart failure, renal failure, etc.; (2) history of psychiatric disorder; (3) those participating in similar trials. Throughout the study, strict adherence to the latest version of the Declaration of Helsinki [15] was maintained. The study has completed the ethical audit of the First Affiliated Hospital of Chengdu Medical College (NO:2021CYFYIRB-BA−69−01). For this study, we used the empirical sample size estimation method, which required at least five to ten times the number of independent variables. The number of independent variables in this survey was 35 items, calculated to be 193 to 365 cases when 10% of questionnaires were invalid.

## Measurements

### Social-demographic questionnaire

We designed the questionnaire, which included gender, age, marital status, place of residence, level of education, working status, monthly household income per capital, duration of illness due to RA, pain, and other factors.

### Bristol Multidimensional Rating Scale for Fatigue in Rheumatoid Arthritis (BRAF-MDQ)

The scale used in the present study was a Chinese scale developed by Hewlett S et al. [16] in 2010 and later translated into Chinese by Gao Lei et al. [17]. The scale consists of somatic dimensions (4 items), life dimensions (7 items), cognitive dimensions (5 items), and affective dimensions (4 items), with a total of 20 items in four dimensions and a total score ranging from 0 to 70, with higher scores indicating greater levels of fatigue. This yielded a total Cronbach’s α of 0.956.

### Short Form Health Survey−12 (SF−12)

Shou Juan et al. [18] used the Chinese-translated version of the SF−12 Health Survey Short Form. The scale has eight dimensions and twelve entries: general health GH (1 entry), physical function PF (2 entries), physical function RP (2 entries), somatic pain BP (1 entry), vitality VT (1 entry), social function SF (1 entry), emotional function RE (2 entries) and MCS MH (2 entries). The total physical health score (PCS) can be calculated using the GH, PF, RP, and BP dimension scores, whereas the total mental health score (MCS) can be calculated using the VT, SF, RE, and MH dimension scores. The scores on each dimension of the scale are converted to a standardized score using the formula [19], with a total score ranging from 0 to 100 and higher scores indicating a higher level of QOL for the patient. This resulted in a total Cronbach’s α of 0.828.

### A Chinese version of the 10-item Connor-Davidson Resilience Scale (CD-RISC−10)

In order to analyze and improve the scale, Campbell-Sills et al. al [20] used the Connor-Davidson Resilience Scale [21] (CD-RISC), which was translated by Chinese scholars Cheng et al. [22]. The scale has three dimensions: resilience, self-enhancement, and optimism, with a total score ranging from 0 to 40, with higher scores indicating high resilience, resulting in a total Cronbach’s α coefficient of 0.862.

### A Chinese version of the Arthritis Self-Efficacy Scale−8-entry version (ASES−8)

In the present study, we used an abbreviated version of the Arthritis Self-Efficacy Scale (ASES) developed into eight items by Lorig et al. [23] in 1989, which our scholar Gao et al. [24] has translated. All items are rated on a scale from 1 to 10, and the total score is the mean of all items, with higher scores indicating a greater sense of self-efficacy, resulting in a total Cronbach’s α of 0.890.

### Data collection

A consistent electronic questionnaire was used to collect data for this study. Questionnaires were completed anonymously. A consistent guideline was used to indicate how to complete the survey, precautions, and sign the informed consent form before completing the survey. Questionnaire quality control included the following items: (1) team members involved in distributing the questionnaire were uniformly trained and strictly included in the study population; (2) all entries in the questionnaire were mandatory and could not be submitted until they were completed; (3) after collecting the questionnaire, the researcher should logically check the data and eliminate data with obvious trends. This study included 423 questionnaires, and 423 were returned, yielding a 100% questionnaire return rate.

### Statistical analysis

For data processing analysis, IBM SPSS 26.0 software was used. Bootstrap with process macros was used to construct and test chain-mediated model effects. The sample correction was repeated 5000 times to examine the significance of the mediation effect by computing 95% confidence intervals for the mediation effect; direct mediation, indirect mediation, or chained mediation effects were considered significant when the 95% confidence interval did not include 0.

## Results

### Common Method Bias (CMB) control and test

Since the data for this study were all derived from subjective questionnaires, a common method bias test was required. In this study, unrelated principal component factor analysis was conducted on 50 entries for all variables using the Harman single-factor test. A total of eight factors were found to have eigenvector values greater than one, and the maximum amount of factor variance accounted for 35.92% of the variance, which was less than the 40% critical value, indicating that there was not a significant common method bias in the present study.

### Participants’ basic demographic characteristics

The study sample included 423 patients with rheumatoid arthritis (79.7% females). They are mostly married (93.4%), have a low level of education (60.8%), and mostly live in urban areas (65.2%) (Table 1).

**Table 1:** The participants characteristics

Variables	*n*	%
**Gender**		
Men	86	20.33
Women	337	79.67
**Age (years)**		
<60	278	65.72
≥ 60	145	34.28
**Marital status**		
Married	395	93.38
Single	13	3.07
Divorced	15	3.55
**Residence**		
Urban	276	65.25
Rural	147	34.75
**Way of living**		
Living alone	78	18.44
With their children	214	50.59
With your spouse	131	30.97
**Educational level**		
Less than a high school graduate	257	60.76
High school	99	23.40
Junior college	44	10.40
Bachelor’s degree and above	23	5.44
**Payment for treatment**		
Medical Insurance	319	75.41
Completely self-funded	104	24.59
**Occupational status**		
Employed (including freelance, self-employed, etc.)	135	31.91
Retirement	132	31.21
Unemployed	156	36.82
**Per capital monthly household income (¥)**		
< 3000	169	39.95
3000 ~ 5000	189	44.68
> 5000	65	15.37
**Disease duration (years)**		
< 1	70	16.55
1 ~ 5	156	36.88
5 ~ 10	81	19.15
10 ~ 20	70	16.55
≥ 20	46	10.87
**Commodities**		
No	302	71.39
Yes	121	28.61
**Articular deformation**		
Yes	180	42.55
No	243	57.45
**Current biologic preparations use**		
Yes	183	43.28
No	240	56.72
**Pain**		
Mild pain	175	41.37
Medium pain	142	33.57
Severe pain	106	25.16
**Functional classifications of joints**		
Level 1	144	34.04
Level 2	236	55.79
Level 3	40	9.46
Level 4	3	0.71

Results reported as n (%) unless otherwise stated

### Correlation analysis of the variables

The correlations between fatigue and resilience, self-efficacy, PCS, and MCS were statistically significant (*r* =−0.56 ~−0.28, *p* < 0.01). The correlations between resilience and self-efficacy, PCS, and MCS were statistically significant (*r* =−0.22 ~ 0.36, *p* < 0.01), whereas the correlations between self-efficacy, PCS, and MCS were statistically significant (*r* = 0.25 ~ 0.41, *p* < 0.01) (Table 2).

**Table 2:** Correlation analysis of the variables

Variables	${\bar x}$ ± s	Fatigue	psychological resilience	Self-efficacy	PCS	MCS
Fatigue	22.65 ± 14.54	1				
psychological resilience	27.41 ± 8.12	-0.28**	1			
Self-efficacy	45.92 ± 16.36	-0.32**	0.26**	1		
PCS	24.52 ± 6.00	-0.35**	0.22**	0.25**	1	
MCS	56.72 ± 11.33	-0.56**	0.36**	0.41**	-0.00**	1

**P<0.01

${\bar x}$ ± s: mean value ± standard deviation; *PCS* physical component summary; *MCS* mental component summary; *P* P Value

### Regression analysis of the variables

Fatigue had a statistically significant predictive effect on resilience, self-efficacy, PCS, and MCS (B =−0.43 ~−0.27, *P* < 0.001). Resilience had a statistically significant predictive effect on self-efficacy, PCS, and MCS (B = 0.12 ~ 0.19, *P* < 0.001), whereas self-efficacy had a statistically significant effect on PCS and MCS (B = 0.22 ~ 0.13, *P* < 0.001) (Table 3).

**Table 3:** Regression analysis of the variables

Dependent variables	Predicted variables	*R* ^2^	Adjusted *R*^2^	*F*	*B*	*t*
Psychological resilience	Fatigue	0.28	0.08	35.17***	-0.28	-5.94***
Self-efficacy	Fatigue	0.37	0.14	33.25***	-0.27	-5.71***
	Psychological resilience				0.19	4.01***
PCS	Fatigue	0.46	0.21	36.94***	-0.27	-5.67***
	psychological resilience				0.12	2.34***
	Self-efficacy				0.13	2.70***
MCS	Fatigue	0.52	0.28	52.97***	-0.43	-10.59***
	Psychological resilience				0.18	4.51***
	Self-efficacy				0.22	5.49***

***P<0.001;

R^2^: Coefficient of Determination; Adjusted R^2^: Adjusted Coefficient of Determination; *F* variance; *B* regression coefficient; *t* student’s t test; *PCS* physical component summary; *MCS* mental component summary; *P* P Value

### Chain-mediated effects test

**Fig. 1:**
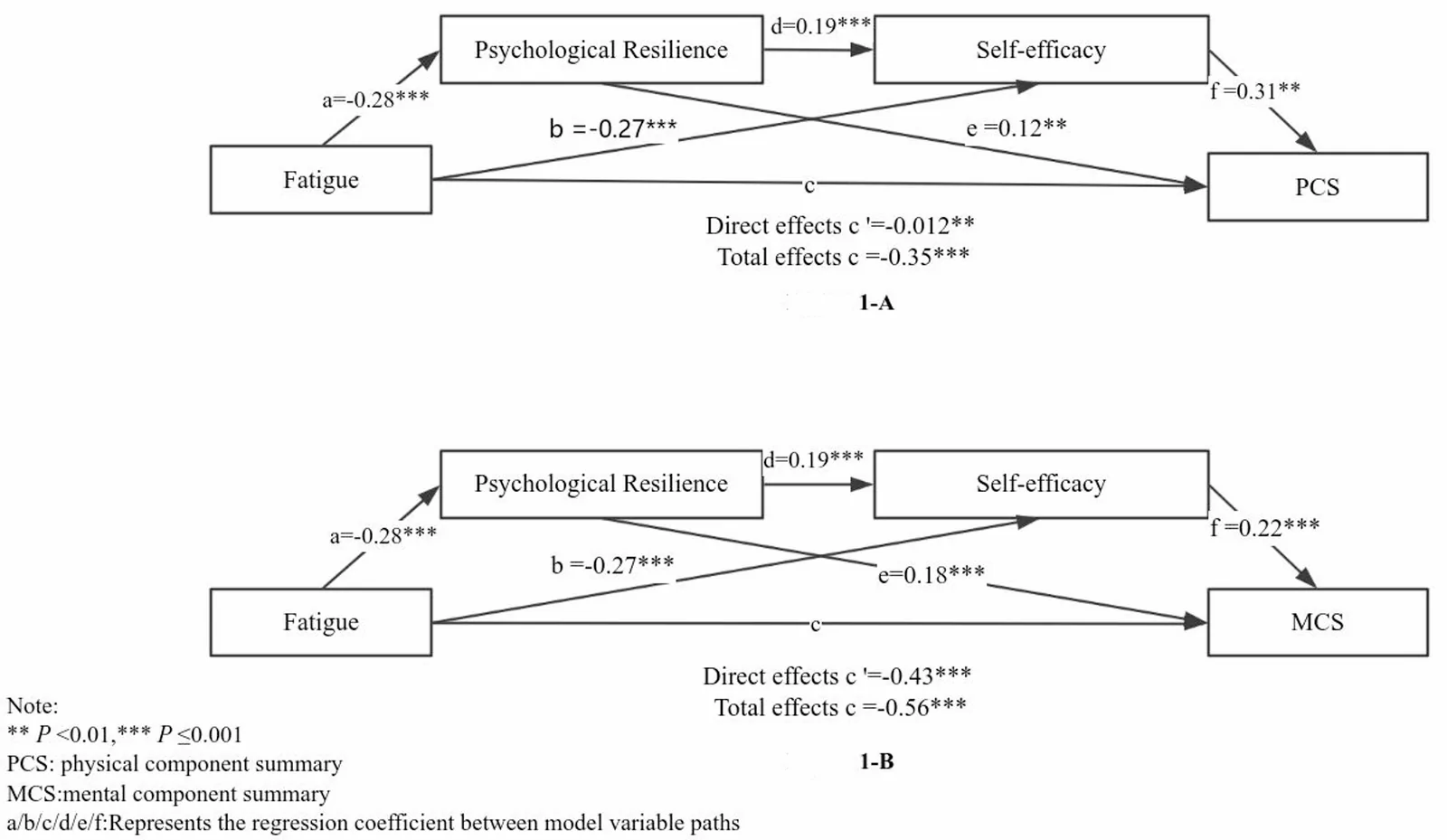
Chain mediation effect model diagram

A 95% CI for the chained mediating effects was estimated using fatigue as an independent variable, somatic and MCS dimensions of QOL as dependent variables, resilience and self-efficacy as mediated variables, a macroprogramming process, and bias correction of non-parametric percentile-guided confidence intervals. The bootstrap 95% confidence intervals for the mediation effects of resilience and self-efficacy did not contain zero, indicating that resilience and self-efficacy play an important mediating chain effect between the dimensions of fatigue and PCS (Table 4). The bootstrap 95% confidence intervals for the mediation effect of resilience and self-efficacy do not contain zero, showing that resilience and self-efficacy have a strong mediating chain effect on both fatigue and MCS dimensions (Table 5). Furthermore, the confidence intervals for the differences between path 1 and path 3 and path 2 and path 3 did not contain a 0 value, indicating that path 1 and path 3 and path 2 and path 3 differed significantly, validating the hypotheses of this study along with Fig. 1.

**Table 4:** PCS chain mediation effect test

Paths	Effect value	SE	95% CI	Percentage of relative impact
Path 3: Fatigue →psychological resilience→Self-efficacy→PCS	-0.007	0.003	-0.014 to -0.001	2.01%
Path 2: Fatigue→psychological resilience→PCS	-0.031	0.017	-0.068 to -0.001	8.88%
Path 1: Fatigue→Self-efficacy→PCS	-0.035	0.016	-0.070 to -0.007	10.03%
Compare 1	-0.349	0.155	-0.693 to -0.091	-
Direct effects	-0.012	0.006	-0.025 to -0.002	78.80%
Total effects	-0.349	0.019	-0.181 to -0.107	-

Compare 1: The difference between Path 1 and Path 3

*SE* Standard Error; *95% CI* 95%Confidence interval; *PCS* physical component summary

**Table 5:** MCS chain mediation effect test

Paths	Effect value	SE	95% CI	Percentage of relative effect
Path 3: Fatigue→psychological resilience→Self-efficacy→MCS	-0.012	0.004	-0.017 to -0.003	2.16%
Path 2: Fatigue→psychological resilience→MCS	-0.060	0.012	-0.065 to -0.017	10.79%
Path 1: Fatigue→Self-efficacy→MCS	-0.050	0.013	-0.075 to -0.024	8.99%
Compare 1	-0.030	0.012	-0.055 to -0.007	-
Compare 2	-0.038	0.012	-0.064 to -0.017	-
Direct effects	-0.434	0.032	-0.401 to -0.276	78.06%
Total effects	-0.556	0.032	-0.496 to -0.372	-

Compare 1: The difference between Path 2 and Path 3; Compare 2: The difference between Path 1 and Path 3

*SE* Standard Error; *95% CI* 95%Confidence interval; *MCS* mental component summary

## Discussion

In this study, patients with RA had a total fatigue level score of (22.65 ± 14.54), which was lower than the findings by Shen et al. [6], most likely due to the good standard of medical care and healthcare facilities in the city where this study was carried out. The majority of RA patients were aware of their level of fatigue, and the population under examination in this study was 55.06 ± 0.48 years old, which was a clear indication of fatigue symptoms. The resilience score of RA patients was (27.41 ± 8.12), which was moderate and higher than the findings of Ye et al. [25]. The reason could be 36.9% of the study population had the disease for 1 to 5 years, and the disease was still at a controllable stage; thus, the psychological burden of the patients was low. For example, RA patients had a self-efficacy score of (45.92 ± 16.36), which was at an intermediate level, similar to the results of Zhao et al [26]. However, self-efficacy of RA patients is primarily influenced by their ability to manage illness. RA patients cannot engage in effective long-term self-management due to long disease duration, recurrent disease, and high financial burden. The scores for the PCS and MCS used in this study were (24.52 ± 6.00) and (56.72 ± 11.33), respectively, with somatic dimension scores significantly lower than the national norm for the general population, consistent with the findings of Bai et al. [27], indicating that the self-management capacity of RA patients is severely affected by the disease. In this study, RA patients had moderate ratings of fatigue, resilience, and self-efficacy, with some MCS problems and generally low ratings of QOL, indicating that there is much room for development in health promotion for RA patients and that the QOL level of RA patients and the factors affecting it deserve the critical attention of medical staff.

However, there was a significant correlation between fatigue and QOL among RA patients (*P* < 0.01), which is consistent with the findings of Yoshii et al. [28] and Tański et al. [29], further confirming that fatigue has a significant impact on the QOL of RA patients. The results of this study are consistent with those of other researchers. Fatigue is a more common symptom in RA patients, characterized by uncontrollable and unrelieved rest [30], which leads to reduced ability to live, negative emotions, impaired work capacity, and ultimately reduced QOL for patients. According to Papa et al. [31], fatigue in RA patients is linked to pain, disease activity, mood, and other factors, which can be treated and managed by themselves in several ways, including active psychological interventions, the development of regular exercise programs, and positive thought therapy. For this reason, the level of fatigue should be actively evaluated in the clinical management of RA patients [32], and treatment plans should be implemented to facilitate negative psychological emotions, reduce fatigue, and improve QOL.

It was found that fatigue in RA patients significantly mediated QOL somatic health and MCS dimensions over resilience (95% CI−0.068,−0.001 and−0.065,−0.017, respectively, with total effect mediated by 8.88% and 10.79%, respectively), i.e., fatigue acted on QOL somatic health and MCS dimensions by 8.88% and 10.79% acted through resilience, which is similar to the findings of Liu et al. [33]. As a positive psychological resource, resilience helps patients flexibly choose coping strategies tailored to their specific needs [34] and mitigates the impact of diverse stressors on their physical and MCS. Conversely, RA patients with higher levels of fatigue may cope negatively with the disease due to recurrent disease and lack of knowledge about the disease, which leads to anxiety and depression, as well as impulsive behavior, resulting in low levels of resilience and a consequent poorer ability to cope with stress, which in turn affects the QOL. Clinical work allows medical staff to assess resilience as part of treatment [35], as well as actively explore methods and strategies for increasing patients’ confidence and initiative, improving resilience, reducing fatigue, and promoting health through measures such as stepwise health education, encouragement to participate in social activities, and flexibility training [10].

The results of the pathway analysis showed that fatigue in RA patients significantly mediated self-efficacy on QOL somatic health and MCS dimensions (95% CI−0.070,−0.007 and−0.075,−0.024, respectively, with total effect mediation of 10.03% and 8.99%, respectively), i.e., fatigue acted on QOL somatic health and MCS dimensions by 10.03% and 8.99% acted through self-efficacy, which is similar to the results of Suh et al. [12] The purpose of this study was to assess the impact of the intervention. Patient self-efficacy is a determinant of patients’ intentions and behaviors, a key protection for controlling patients’ illness, and is associated with factors such as fatigue and psychological mood [36]. More fatigued RA patients were less compliant and motivated to get treated [37], leading to lower self-efficacy, negative mood, and poorer QOL. Improving self-efficacy in RA patients is primarily associated with increased physical activity and improved drug compliance [37]. Personalized intervention programs are developed through health education to improve patient self-management, self-efficacy, and healthy behavior, thereby improving patient disease outcomes.

In summary, the results of this study indicate that resilience and self-efficacy in people with RA play a chain mediating effect between fatigue and both the somatic and MCS dimensions of QOL (95% CI−0.014,−0.001 and−0.017,−0.003 respectively, accounting for 2.01% and 2.16% of the total effect values). Patients with RA who had higher levels of fatigue were less able to psychologically rebound while facing stress, affecting their ability to self-manage their illness and, in turn, their QOL. Conversely, RA patients with less fatigue may gain new resilience amid constant challenges [7] and have a correspondingly higher level of resilience, greater competence and confidence in disease management, and a higher QOL. Psychologically resilient RA patients are better able to manage the negative emotions associated with their condition [37], decide to accept and be open in the face of stress, and typically have higher levels of self-efficacy. As a contributor to patients’ QOL, self-efficacy may also help RA patients to manage disease progression, reduce fatigue, and achieve effective self-management. Thus, when managing illness and improving the QOL in RA patients, the assessment of the level of resilience and self-efficacy should be considered an important component of chronic disease management to increase patients’ confidence in coping with illness, enhance their ability to manage chronic illness and improve their QOL.

## Strengths and limitations

The study has several advantages. First, a large number of RA patients were recruited through a multicenter routine clinical practice setting where patients were recruited continuously with a low risk of selection bias, and the results were rather generic. Furthermore, we implemented strict quality control during the study to ensure the validity and reliability of the results.

The present study has some limitations. First: this is a cross-sectional study that estimates only correlations between variables rather than accurately explaining cause-and-effect relationships between fatigue, resilience, self-efficacy, and QOL. Second, all data were obtained from questionnaires reported by patients, which may be subjective or susceptible to recall bias.

## Clinical implications

This study shows that fatigue in RA can directly or indirectly affect their QOL through the mediating effects of psychological elasticity, self-efficacy, and the interlocking mediating effects of psychological elasticity. In order to effectively improve the QOL of RA patients, any type of fatigue, resilience, and self-efficacy must be addressed. Therefore, patients’ fatigue, resilience, and self-efficacy should be actively assessed in clinical practice [32]. Active mindfulness therapy and resilience training should boost patient confidence in disease response and improve chronic disease management as well as QOL.

## Conclusions

This study aimed to build a chain mediation model to analyze the mechanism of fatigue on QOL in RA patients. Resilience and self-efficacy were found to form a chain-mediated relationship between fatigue and QOL in people with RA. In summary, the results of this study provide a theoretical foundation for how to effectively improve the QOL of RA patients. Furthermore, the findings imply that when it comes to improving the QOL of people with RA, employees should focus on their resilience and self-efficacy in addition to their level of fatigue. Individualized interventions can be implemented for people with RA based on assessing fatigue, resilience, and self-efficacy to improve their capacity for self-management and QOL.

## Data Availability

Due to the necessity to safeguard patient privacy and maintain the moral management of hospital research, the data utilized in this study are not publicly accessible on the Internet. But, if you have a worthwhile reason, you can get the information by getting in touch with the corresponding author.

## References

[1] van der Heijde D, Kartman CE, Xie L, et al. Radiographic progression of structural joint damage over 5 years of Baricitinib treatment in patients with rheumatoid arthritis: results from RA-BEYOND. J Rheumatol. 2022;49:133–41. 34526397 10.3899/jrheum.210346

[2] De Cock D, Doumen M, Vervloesem C, et al. Psychological stress in rheumatoid arthritis: a systematic scoping review. Semin Arthritis Rheum. 2022;55:152014. 35489168 10.1016/j.semarthrit.2022.152014

[3] Cramp F. The role of non-pharmacological interventions in the management of rheumatoid-arthritis-related fatigue. Rheumatology (Oxford). 2019;58:v22–8. 31682276 10.1093/rheumatology/kez310 PMC6827265

[4] Fernandez-Gonzalez M, Fernandez-Lao C, Martin-Martin L, et al. Therapeutic benefits of Balneotherapy on Quality of Life of patients with rheumatoid arthritis: a systematic review. Int J Environ Res Public Health. 2021;18:13216. 34948827 10.3390/ijerph182413216 PMC8701266

[5] Alvarez MC, Albuquerque M, Neiva HP, et al. Exploring the relationship between Fibromyalgia-related fatigue, physical activity, and Quality of Life. Int J Environ Res Public Health. 2022;19:13216. 35457737 10.3390/ijerph19084870 PMC9032824

[6] Shen B, Chen H, Yang D, et al A structural equation model of Health-related quality of life in Chinese patients with rheumatoid arthritis. Front Psychiatry. 2021;12:716996. 34421688 10.3389/fpsyt.2021.716996 PMC8371236

[7] Shaw Y, Bradley M, Zhang C, et al. Development of Resilience among Rheumatoid Arthritis patients: a qualitative study. Arthritis Care Res (Hoboken). 2020;72:1257–65. 31282121 10.1002/acr.24024

[8] Chan SW. Chronic Disease Management, Self-Efficacy and Quality of Life. J Nurs Res. 2021;29:e129. 33427791 10.1097/JNR.0000000000000422 PMC7808345

[9] Guerrini UA, Varallo G, Granese V, et al. The impact of psychological flexibility on Psychological Well-being in adults with obesity. Front Psychol. 2021;12:636933. 33828505 10.3389/fpsyg.2021.636933 PMC8019785

[10] Majnarić LT, Bosnić Z, Guljaš S, et al. Low psychological resilience in older individuals: an Association with increased inflammation, oxidative stress and the Presence of Chronic Medical conditions. Int J Mol Sci. 2021;22:8970. 34445675 10.3390/ijms22168970 PMC8396457

[11] Bandura A. Self-efficacy: toward a unifying theory of behavioral change. Psychol Rev. 1977;84:191–215. 847061 10.1037//0033-295x.84.2.191

[12] Suh CH, Lee K, Kim JW, Boo S. Factors affecting quality of life in patients with rheumatoid arthritis in South Korea: a cross-sectional study. Clin Rheumatol. 2022;41:367–75. 34609663 10.1007/s10067-021-05944-9

[13] Zhang J, Wang X, Xu T, et al. The effect of resilience and self-efficacy on nurses’ compassion fatigue: a cross-sectional study. J Adv Nurs. 2022;78:2030–41. 34825731 10.1111/jan.15113

[14] Aletaha D, Neogi T, Silman AJ, et al. 2010 rheumatoid arthritis classification criteria: an American College of Rheumatology/European League against Rheumatism collaborative initiative. Ann Rheum Dis. 2010;69:1580–8. 20699241 10.1136/ard.2010.138461

[15] World Medical Association Declaration. Of Helsinki: ethical principles for medical research involving human subjects. JAMA. 2013;310:2191–4. 24141714 10.1001/jama.2013.281053

[16] Hewlett S, Dures E, Almeida C. Measures of fatigue: Bristol rheumatoid arthritis fatigue multi-dimensional questionnaire (BRAF MDQ), Bristol rheumatoid arthritis fatigue Numerical Rating scales (BRAF NRS) for severity, effect, and coping, chalder fatigue questionnaire (CFQ), Checklist Individual Strength (CIS20R and CIS8R), fatigue severity scale (FSS), Functional Assessment Chronic illness therapy (fatigue) (FACIT-F), Multi-dimensional Assessment of fatigue (MAF), multi-dimensional fatigue inventory (MFI), Pediatric Quality of Life (PedsQL) multi-dimensional fatigue Scale, Profile of fatigue (ProF), short form 36 vitality Subscale (SF–36 VT), and Visual Analog scales (VAS). Arthritis Care Res (Hoboken). 2011;63(Suppl 11):S263–86. 22588750 10.1002/acr.20579

[17] Gao L, Sun Y, Pan L, et al. Current status and influencing factors of fatigue in patients with rheumatoid arthritis: a cross-sectional study in China. Int J Nurs Pract. 2022;28:e12996. 34309127 10.1111/ijn.12996

[18] Shou J, Ren L, Wang H, et al. Reliability and validity of 12-item short-form health survey (SF–12) for the health status of Chinese community elderly population in Xujiahui district of Shanghai. Aging Clin Exp Res. 2016;28:339–46. 26142623 10.1007/s40520-015-0401-9

[19] Ware JJ, Kosinski M, Keller SD. A 12-Item short-form Health Survey: construction of scales and preliminary tests of reliability and validity. Med Care. 1996;34:220–33. 8628042 10.1097/00005650-199603000-00003

[20] Campbell-Sills L, Stein MB. Psychometric analysis and refinement of the Connor-Davidson Resilience Scale (CD-RISC): validation of a 10-item measure of resilience. J Trauma Stress. 2007;20:1019–28. 18157881 10.1002/jts.20271

[21] Connor KM, Davidson JR. Development of a new resilience scale: the Connor-Davidson Resilience Scale (CD-RISC). Depress Anxiety. 2003;18:76–82. 12964174 10.1002/da.10113

[22] Cheng C, Dong D, He J, Zhong X, Yao S. Psychometric properties of the 10-item Connor-Davidson Resilience Scale (CD-RISC–10) in Chinese undergraduates and depressive patients. J Affect Disord. 2020;261:211–20. 31654919 10.1016/j.jad.2019.10.018

[23] Lorig K, Chastain RL, Ung E, et al. Development and evaluation of a scale to measure perceived self-efficacy in people with arthritis. Arthr Rhuem. 1989;32:37–44. 10.1002/anr.1780320107 2912463

[24] Gao L, Zhang XC, Li MM, Yuan JQ, Cui XJ, Shi BX. Psychometric properties of the Chinese version of arthritis self-efficacy Scale–8 (ASES–8) in a rheumatoid arthritis population. Rheumatol Int. 2017;37:751–6. 28063069 10.1007/s00296-016-3640-y

[25] Ye ZJ, Zhang Z, Tang Y, et al. Development and psychometric analysis of the 10-item resilience scale specific to cancer: a multidimensional item response theory analysis. Eur J Oncol Nurs. 2019;41:64–71. 31358259 10.1016/j.ejon.2019.06.005

[26] Zhao WH, Zhang LX, Liu CX, Niedermann K, Yang HZ, Luo W. Validation of the Chinese version of joint protection self-efficacy scale in patients with rheumatoid arthritis. Clin Rheumatol. 2019;38:2119–27. 30972575 10.1007/s10067-019-04510-8

[27] Bai B, Chen M, Fu L, et al. Quality of life and influencing factors of patients with rheumatoid arthritis in Northeast China. Health Qual Life Outcomes. 2020;18:119. 32366246 10.1186/s12955-020-01355-7 PMC7197177

[28] Yoshii I, Sawada N, Chijiwa T. Associations between clinical metrics of joint deformity, disease duration, disease activity, functional capacity, quality of life, pain, and fatigue in patients with rheumatoid arthritis. Clin Rheumatol. 2022;42:1027–38. 36371481 10.1007/s10067-022-06432-4

[29] Tański W, Wójciga J, Jankowska-Polańska B. Association between Malnutrition and Quality of Life in Elderly patients with rheumatoid arthritis. Nutrients. 2021;13:1259. 33921207 10.3390/nu13041259 PMC8070444

[30] Ifesemen OS, McWilliams DF, Norton S, Kiely P, Young A, Walsh DA. Fatigue in early rheumatoid arthritis: data from the early rheumatoid Arthritis Network. Rheumatology (Oxford). 2022;61:3737–45. 34958376 10.1093/rheumatology/keab947 PMC9434222

[31] Pope JE. Management of fatigue in rheumatoid arthritis. Rmd Open. 2020;6:e1084. 10.1136/rmdopen-2019-001084 PMC7299512 32385141

[32] Santos E, Duarte C, Da SJ, Ferreira R. The impact of fatigue in rheumatoid arthritis and the challenges of its assessment. Rheumatology (Oxford). 2019;58:v3–9. 31435662 10.1093/rheumatology/kez351 PMC6827262

[33] Liu L, Xu X, Xu N, Wang L. Disease activity, resilience and health-related quality of life in Chinese patients with rheumatoid arthritis: a multi-center, cross-sectional study. Health Qual Life Outcomes. 2017;15:149. 28738816 10.1186/s12955-017-0725-6 PMC5525274

[34] Ziarko M, Siemiątkowska K, Sieński M, Samborski W, Samborska J, Mojs E. Mental Health and Rheumatoid Arthritis: toward understanding the emotional status of people with chronic disease. Biomed Res Int. 2019;2019:1473925. 30886858 10.1155/2019/1473925 PMC6388315

[35] Ziarko M, Mojs E, Sikorska D, Samborski W. Coping and life satisfaction: mediating role of Ego-resiliency in patients with rheumatoid arthritis. Med Princ Pract. 2020;29:160–5. 31557754 10.1159/000503708 PMC7098290

[36] Zhang Q, Huang F, Zhang L, Li S, Zhang J. The effect of high blood pressure-health literacy, self-management behavior, self-efficacy and social support on the health-related quality of life of Kazakh hypertension patients in a low-income rural area of China: a structural equation model. BMC Public Health. 2021;21:1114. 34112122 10.1186/s12889-021-11129-5 PMC8194055

[37] Peters M, Potter CM, Kelly L, Fitzpatrick R. Self-efficacy and health-related quality of life: a cross-sectional study of primary care patients with multi-morbidity. Health Qual Life Outcomes. 2019;17:37. 30764833 10.1186/s12955-019-1103-3 PMC6376655

